# Superoxide Dismutase1 Levels in North Indian Population with Age-Related Macular Degeneration

**DOI:** 10.1155/2013/365046

**Published:** 2013-11-30

**Authors:** Akshay Anand, Neel K. Sharma, Amod Gupta, Sudesh Prabhakar, Suresh K. Sharma, Ramandeep Singh

**Affiliations:** ^1^Department of Neurology, Postgraduate Institute of Medical Education and Research (PGIMER), Chandigarh, India; ^2^Department of Ophthalmology, Postgraduate Institute of Medical Education and Research (PGIMER), Chandigarh 160012, India; ^3^Department of Statistics, Panjab University, Chandigarh 160014, India

## Abstract

*Aim.* The aim of the study was to estimate the levels of superoxide dismutase1 (SOD1) in patients of age-related macular degeneration (AMD) and examine the role of oxidative stress, smoking, hypertension, and other factors involved in the pathogenesis of AMD. *Methods.* 115 AMD patients and 61 healthy controls were recruited for this study. Serum SOD1 levels were determined by ELISA and were correlated to various risk factors. Logistic regression model of authenticity, by considering SOD1 as independent variable, has been developed along with ROC curve. *Results.* The SOD1 levels were significantly higher in AMD patients as compared to those of the controls. The difference was not significant for wet and dry AMD. However, the difference was significant between wet AMD subtypes. Nonsignificance of the Hosmer-Lemeshow goodness of fit statistic (*χ* ^2^ = 10.516, df = 8, *P* = 0.231) indicates the appropriateness of logistic regression model to predict AMD. *Conclusion.* Oxidative stress in AMD patients may mount compensatory response resulting in increased levels of SOD1 in AMD patients. To predict the risk of AMD on the basis of SOD1, a logistic regression model shows authenticity of 78%, and area under the ROC curve (0.827, *P* = .0001) with less standard error of 0.033 coupled with 95% confidence interval of 0.762–0.891 further validates the model.

## 1. Introduction

AMD is the most important cause of blindness which is characterized by progressive degeneration of macula leading to severe irreversible loss in vision [1]. The vision loss results either from retinal degeneration (dry AMD) or from the choroidal neovascularization (wet AMD). The clinical manifestation of AMD includes drusen, geographic atrophy, hyperplasia of the retinal pigment epithelium (RPE), and angiogenesis of choroidal vessels (CNV) [2].

Smoking, alcohol, oxidative stress, and genetic factors are implicated in the pathogenesis of AMD [3], but the exact cause of AMD remains complex. It has been reported that aging is associated with pathological and biochemical changes in the eye. In general, aging and AMD are believed to result from cumulative and increased oxidative damage [4]. Oxidative stress can exert molecular or cellular damage mediated by reactive oxygen species (ROS) which has been earlier shown to be implicated with diseases of ageing [5]. The elevated levels of endogenously synthesized ROS are known to be regulated by various antioxidant, enzymatic, and nonenzymatic protective biochemical mechanisms like Glutathione peroxidase (GPx), superoxide dismutase (SOD), and catalase (CAT) [6]. ROS which includes free radicals, nascent oxygen, hydrogen peroxide and the by-products of oxygen metabolism are deleterious for eye pathophysiology. Due to the high consumption of O_2_, the high concentration of polyunsaturated fatty acid and direct exposure of light render retina susceptible to oxidative stress [7]. Various factors are responsible for oxidative stress generated from aging; these include decreased levels of vitamin C and vitamin E in plasma [8]. It has also been shown that oxidized glutathione levels increase in plasma and that glutathione levels decrease with the age [9]. Increased lipid peroxidation is also reported in aging [10], and the consequences of these imbalanced biochemical changes lead to increased susceptibility of retinal pigment epithelium cells (RPE) to oxidative damage with aging. Even catalase activity and vitamin E levels have been reported to decrease with aging in RPE cells [11]. There are several pathological features which accompany aging. These include increased volume of lipofuscin contents (increased lipid and protein contents) which enhance the oxidative damage susceptibility and decreased optical density of macular pigment [12] which results in membrane blebbing of RPE cells, a phenomenon observed in AMD eyes and aging [13].

We hypothesized that oxidoreduction alteration in the eye might result from deranged SOD1 levels. We, therefore, analysed the expression of superoxide dismutase1 in patients of AMD as compared to controls. The major antioxidant system in the retina consists of three superoxide dismutase (SOD) isoenzymes that catalyse dismutation of superoxide into oxygen and hydrogen peroxide (H_2_O_2_) [14]. SOD is an antioxidant enzyme useful in the defense system against ROS. Superoxide dismutase catalyzes the dismutation reaction of O_2_^−^ (superoxide radical anion) to H_2_O_2_, which is then catalyzed to H_2_O and O_2_ by catalase and glutathione peroxidase. There are three major families of superoxide dismutase, depending on metal cofactors: Cu-Zn SOD (SOD1), present in cytosol, Mn (Fe)-SOD (SOD2) present in mitochondrial matrix, and the extracellular SOD (SOD3) interstitium of the tissues as a secretary form [15].

The amount and activity of the Cu-Zn SOD (SOD1) are the highest among the three isoenzymes in human retina, so it seems reasonable to screen SOD1 for possible role in accelerating age-linked changes in the retina [15].

Currently, there is no study examining the role of SOD1 in Indian AMD patients, and this investigation will likely provide the substrate for future therapies in AMD.

## 2. Methodology

### 2.1. Study Participants

This study was approved by Institute Ethics Committee of Postgraduate Institute of Medical Education and Research, Chandigarh, India (letter no. Micro/10/1411). Patients and controls were first informed about the study and thereafter enrolled in patient/control group after obtaining written proforma from all participants. All enrolled participants were recruited from the Department of Ophthalmology, PGIMER, Chandigarh, India, in which phenotypic criteria were strictly followed. A retina specialist carried out ophthalmic examination of all AMD patients for best corrected visual acuity, dilated fundus examination, and slit lamp biomicroscopy of anterior segment. All patients underwent optical coherence tomography (OCT) and fluorescein fundus angiography (FFA). AMD diagnosis was based on FFA and ophthalmoscopic findings.

We included a total of 176 case-control samples consisting of 115 AMD patients from PGIMER, Chandigarh, India, with 61 genetically distinct healthy controls as per inclusion and exclusion criteria. However, some demographic details were not available for some subjects.

### 2.2. Inclusion and Exclusion Criteria

50 years or older AMD patients with more than five drusen in case of dry AMD in at least one eye and/or choroidal neovascularization in case of wet AMD were incorporated in the study [16, 17]. The controls in the study included those with age 50 years or more with the absence of other diagnostic criteria for AMD.

The exclusion criteria excluded the retinal diseases involving the outer retinal layers and/or photoreceptors other than AMD loss, such as central serous retinopathy, high myopia, diabetic retinopathy, retinal dystrophies, uveitis, and vein occlusion, or similar outer retinal diseases that have been present earlier to the age of 50 and opacities of the ocular media, or other problems enough to preclude satisfactory stereo fundus photography. These situations contain occluded pupils due to cataracts and opacities and synechiae due to ocular diseases.

### 2.3. Baseline Examination

A trained interviewer collected the information about medical history, demographic characteristics, and lifestyle risk factors like smoking, alcohol, and so forth, using a standard risk factor questionnaire [18, 19]. Smokers were defined as those having smoked at least three cigarettes per day or 54 boxes for at least 6 months and were segregated further into smokers and nonsmokers. Nonvegetarian patients were defined as those having chicken, meat, or fish for at least 6 months, and alcohol consumers were defined as those having whiskey, rum, wine, or homemade alcohol for at least 6 months. Hypertension was defined as diastolic blood pressure ≥90 mm Hg and systolic blood pressure ≥140 mm Hg at the time of examination and for this condition whether they had ever taken medications. Similar practices have been used in previous studies [20]. Participants were also asked to report any previous diagnosis of migraine, use of antihypertensive medications, stroke, diabetes, or history of heart diseases.

### 2.4. Collection of Blood and Serum Separation

4.0 mL of blood was collected in serum separator tube (BD Biosciences, USA) from both AMD and controls and left for 30 minutes at 37°C to allow it to clot according to the standard procedures. Serum was subsequently separated by centrifugation for 30 minutes at 3000 rpm. The separated serum was frozen at −80°C until analysis [21, 22].

### 2.5. Total Protein Estimation

The Bradford assay was used to estimate the total serum proteins for normalization of SOD1 levels analysed by ELISA as per the manufacturer's recommendations [23, 24].

### 2.6. SOD1 Expression

Serum from AMD patients and controls was used to carry out the quantitative detection of SOD1 using commercially available enzyme-linked immunosorbant assay (ELISA) (AB Frontier; Catalog no. LF-EK0101) as per the instructions from manufacturer, and absorbance was taken at 450 nm using 680XR Microplate reader (Biorad, Hercules, USA). Sample assays were carried out in duplicate. The procedure to analyse the SOD1 levels was provided by manufacturer of the kit. This assay recognizes native and recombinant human SOD1 with the detection of more than 12.5 pg/mL. The standard curve was generated by linear regression analysis for SOD1 in both controls and patients. All the values were normalized with total serum protein.

### 2.7. Statistical Analysis

All statistical calculations were carried out by statistical product and service solutions SPSS (IBM SPSS Statistics 20.0, Chicago, IL, USA) software. The assumption of normality was tested with the help of Normal Quantile plot (Q-Q plot), and it was observed that data were not normally distributed. Therefore, the Mann-Whitney *U*-test was applied to compare the two groups. For comparing more than two groups, the Kruskal-Wallis oneway analysis of variance (ANOVA) followed by post hoc for multiple comparisons was applied. The *P* ≤ 0.05 was considered significant. The measure *R* ^2^ (coefficient of determination) was used to determine the goodness of standard curve fit for ELISA and total protein. The linear and quadratic regressions with *R* ^2^ > 0.80 were considered to be of a good fit. In order to identify the risk factors associated with AMD, a logistic regression was carried out, and adjusted odds ratios were also obtained. ROC (receiver operating characteristics) curve defines the sensitivity/specificity of the experiment. The ROC curve is basically important for the evaluation of diagnostic tests. The true positive rate (sensitivity) is plotted as the function of the false positive rate (100-specificity) for different cut-off points of a parameter. Each point on the ROC curve represents a sensitivity/specificity pair corresponding to a particular decision threshold. The area under the ROC curve (AUC) is a measure of how well a parameter can distinguish between two diagnostic groups (diseased/normal). ROC curve for predicted model was mapped [25, 26].

## 3. Results

**Figure 1:**
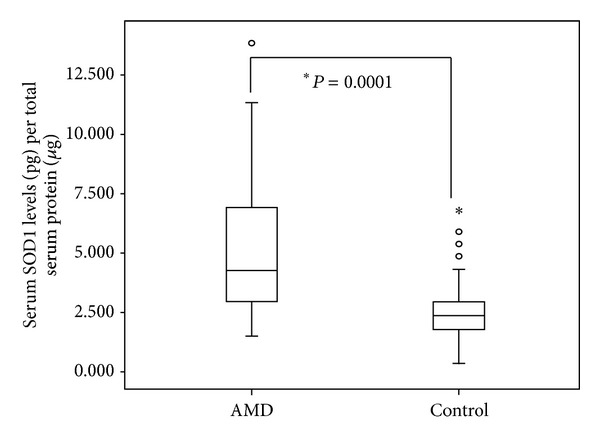
Serum levels of SOD1 in AMD and normal controls. Boxes include values from the first quartile (25th percentile) to third quartile (75th percentile). The thick horizontal line in the box represents median for each dataset. Outliers and extreme values are shown in circles and asterisk, respectively. Levels of SOD1 were normalized to total protein. Data was analyzed by using the Mann-Whitney *U* test.

**Figure 2:**
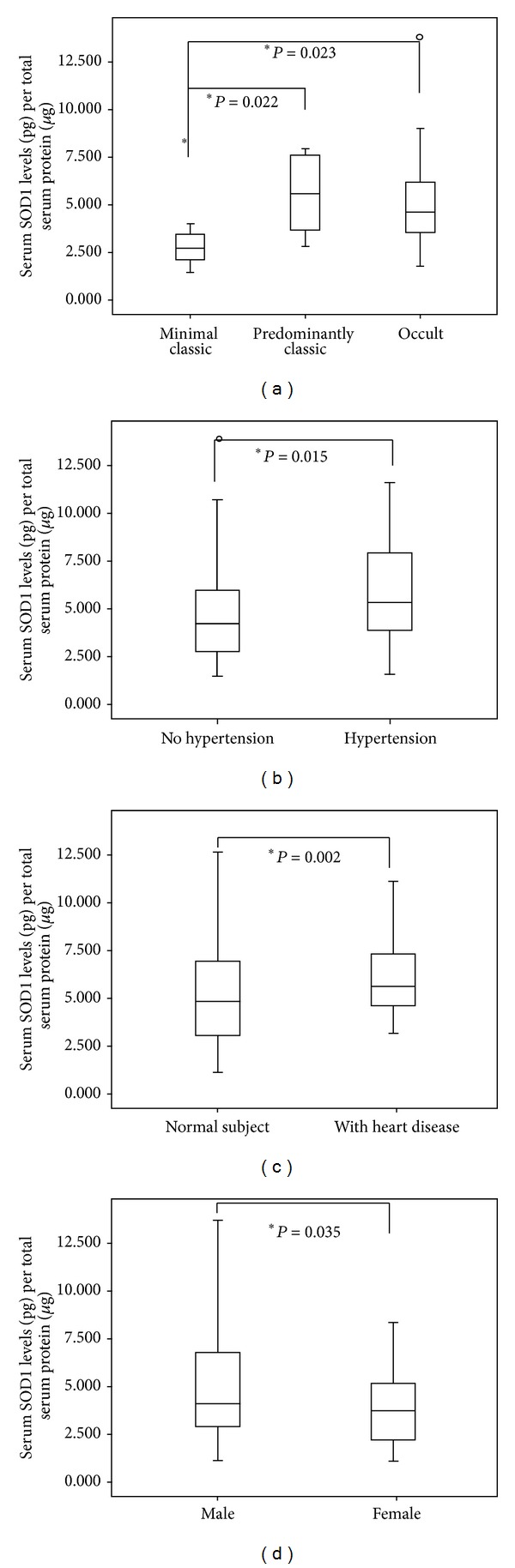
(a) Serum levels of SOD1 in minimal classic, predominant classic, and occult AMD. (b) Serum levels of SOD1 in hypertensive and nonhypertensive AMD patients. (c) Serum levels of SOD1 in heart disease and no heart disease patients. (d) Serum levels of SOD1 in male and female AMD patients. Boxes include values from the first quartile (25th percentile) to the third quartile (75th percentile). The thick horizontal line in the box represents median for each dataset. Outliers and extreme values are shown in circles and asterisks, respectively. Levels of SOD1 were normalized to total protein. Data was analyzed by using the Mann-Whitney *U* test.

**Figure 3:**
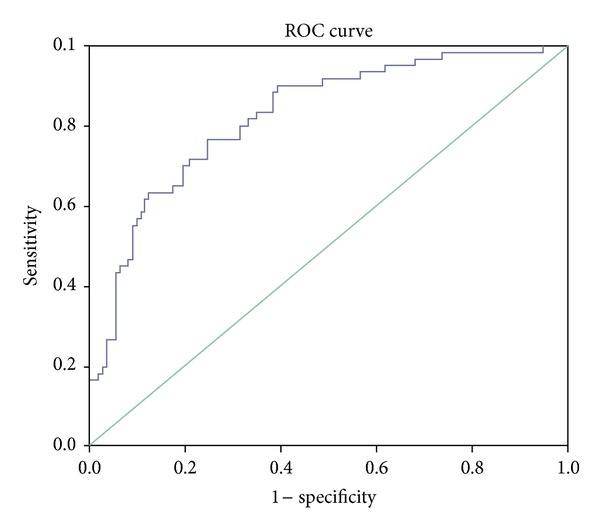
Receiver operating characteristic (ROC) obtained from binary logistic regression model which generates significant predictors of AMD. Area under the curve is reported to be 82.7%.

Summary statistics of important variables have been shown in Table 1. 115 AMD patients were recruited for the study with average age of 64.97 ± 7.1, whereas 61 controls were recruited with an average age of 60.38 ± 13.2. The AMD population was divided according to presence of clinical features and Avastin treatment. The recruited patients and controls were further classified based on their food habits and smoking as well as alcohol consumption and the presence of other associated diseases like hypertension, heart disease, and so forth in order to estimate the levels of SOD1 among these groups. The serum SOD1 level was found to be significantly elevated in AMD subjects as compared to normal controls (Figure 1, Table 2, *P* = 0.0001). However, there was no significant difference between the levels of dry and wet AMD (Table 2, *P* = 0.117). Moreover, in the wet AMD subgroups, significant difference was found. The levels of SOD1 in predominantly classic (*P* = 0.022) and occult AMD patients (*P* = 0.023) were significantly higher as compared to those of minimal classic (Figure 2(a)). An independent analysis was carried out while adjusting the risk factors to AMD. Important risk factors like smoking, alcohol, food habits, gender, hypertension, and heart diseases were analyzed to examine their association with SOD1. The SOD1 levels were found to be higher among hypertensive patients (Figure 2(b), Table 2, *P* = 0.015), those with heart disease (Figure 2(c), Table 2, *P* = 0.002) and male AMD patients (Figure 2(d), Table 2, *P* = 0.035), as compared to nonhypertensive patients or those without heart disease and female AMD patients, respectively. However, the difference was not significant between AMD smokers and AMD nonsmokers, alcohol consumers and alcohol nonconsumers, and vegetarian and nonvegetarians (Table 2). The levels were not found to be significant when compared among Avastin treated AMD patients versus untreated AMD patients (Table 2). It has been observed that there was a significant association of levels of SOD1 with AMD subtypes (*χ* ^2^ = 6.326, *P* = .042), gender (*χ* ^2^ = 6.860, *P* = .032), and smoking (*χ* ^2^ = 6.291, *P* = .043). The prediction equation for AMD, by considering SOD1 as independent variable, shows that 78% of the cases have been correctly classified (model authenticity 78%) with attending confidence intervals for ROC curve. The area under ROC was 0.827 (*P* = .0001) with standard error of 0.033 and confidence interval of 0.762–0.891 (Figure 3).

## 4. Discussion

The major reason for vision loss in elderly population is accounted for by AMD [27]. To understand the mechanism of AMD pathogenesis, several studies have attempted to correlate various targets and biomarkers with conflicting reports and unverified data from the Caucasian population. Facts suggest that oxidative stress plays a major role in the pathogenesis of AMD [28, 29].

This study was carried out to determine whether the serum SOD1 levels are altered in AMD patients as compared to normal controls as this region is characterized by unique dietary habits. Results from this study indicate that the SOD1 levels were elevated significantly in AMD as compared to normal controls. To our knowledge, this non-Caucasian study is first to demonstrate elevated SOD1 serum levels in Indian AMD patients. However, several other studies have been carried out to estimate the activity (not levels) of SOD along with other biomarkers associated with oxidative stress in various population [30–32].

Retina is very susceptible for lipid peroxidation [11, 12] which increases with age in macular region [11]. This is associated with cellular damage which involves decreased cellular antioxidants [13]. In our results, the high levels of SOD1 indicate that lipid peroxidation and oxidative stress are involved in tissue damage in AMD patients. Whether increased SOD1 levels in our study are indeed due to compensatory regulation or causative of AMD can be determined by conducting longitudinal study performed on intermediate or early AMD patients.

SOD1 levels of occult and predominantly classic AMD patients were found to be higher as compared to those of minimally classic AMD patients. This corresponds to disease severity whether induced by SOD1 or resulting from disease. Interestingly, it has been shown previously that the protein content of SOD1 and SOD2 in RPE homogenates increases in the later stages of AMD [33].

The fact that SOD1 levels were found to be higher among male, hypertensive, and heart disease patients could be ascribed to high oxidative stress in these patients. It was shown that the oxidative stress could be involved in the cardiovascular diseases and hypertensive patients [34, 35]. It is pertinent to point out that although SOD1 is an antioxidant, its over expression can lead to increased oxidative stress. Studies on transgenic animals have shown that increased levels of SOD1 lead to more hypersensitivity to oxidative stress [36, 37]. It is possible that the negative effects seen with high levels of SOD1 are caused by an increased level of the product of the dismutation reaction which yields hydrogen peroxide [38]. Kowald et al. have defined such situation by deriving mathematical equations and proposed the three alternative mechanisms driven by SOD1: (i) reaction of hydrogen peroxide with CuZnSOD that leads to formation of hydroxyl radicals, (ii) superoxide radicals acting as chain breaker, and (iii) interchange between oxidized and reduced form of SOD while detoxifying superoxide radicals [38]. These studies do not demonstrate the dual function of SOD, but instead they indicate an alternative pathway which is driven by the free radical burden inside the cell. Recently, it has been found that peroxidase activity of superoxide dismutase depends on CO_2_. The generation of free radicals by the peroxidase activity of superoxide dismutase was found to be higher in the presence of bicarbonate-carbon dioxide. This mechanism explains why strong oxidant is generated during peroxidase activity of SOD which is followed by CO_2_ oxidation to carbonate radicals. These free carbonate radicals have tendency to oxidize various biomolecules inside the cell [39].

Moreover, we have earlier reported that the VEGFR2 levels increased significantly in the AMD patients as compared to those in normal control [40]. We hypothesized that there is positive correlation between the increased SOD1 and VEGFR2 levels because under *in vitro* conditions oxidative stress has been correlated previously with upregulation of VEGF and is thought to be involved in the increased expression of VEGF [41, 42]. In addition, several studies involving tissue culture and animal models have pointed out that oxidative stress is a critical moderator in the transduction of the mitogenic effects of VEGF [43, 44].

We have attempted to predict AMD based on SOD1 using logistic regression, which showed 78% model predictivity, and area under curve is 0.827. The high value of AUC may be used to diagnose AMD patients with very less standard error. The association with gender, smoking, and AMD types means that the increased levels of SOD1 are associated with these factors.

Therefore, the oxidative stress is considered as an important causative factor for AMD, which can lead to induced apoptosis of RPE and result in impairment of RPE function [45–47], and, hence, its analysis in larger AMD cohort is imperative.

## Figures and Tables

**Table 1:** Clinical and demographic details of subjects.

Variables	AMD	Controls
Total	115	61
Wet AMD	84 (73.04%)	—
Dry AMD	31 (26.96%)	—
Minimal classic	7 (11.9%)	—
Predominant classic	16 (27.1%)	—
Occult	36 (61.0%)	—
Avastin treated	55 (65.5%)	—
Not treated with Avastin	29 (34.5%)	—
Duration of disease^¥^	23 ± 2.6 (M)	—
Smokers	50 (43.5%)	11 (20%)
Nonsmokers	65 (56.5%)	44 (80%)
Alcoholic	37 (32.2%)	17 (30.9%)
Nonalcoholic	78 (67.8%)	38 (69.1%)
Vegetarian	61 (53%)	31 (56.4%)
Nonvegetarian	54 (47%)	24 (43.6%)
Hypertension	52 (45.2%)	10 (16.4%)
Nonhypertensive	61 (53%)	45 (73.8%)
Heart disease	16 (13.9%)	—
No heart disease	60 (52.2%)	55 (100%)
Age	64.97 ± 7.1	60.38 ± 13.2
Male	75 (65.2%)	40 (65.6%)
Female	40 (34.8%)	21 (34.4%)

AMD: age-related macular degeneration; M: months; Age: age of onset. Values are mean ± SD or percentage, ^*¥*^Duration of disease is the interval between appearance of the first symptom of AMD and collection of sample. AMD subjects were asked to provide all clinical and demographic details at the age of disease onset.

**Table 2:** SOD1 levels according to different subtypes. ELISA levels were compared by applying the nonparametric Kruskal-Wallis *H* test followed by the Mann-Whitney *U* test.

Subjects	Mean rank	*Z* value	*P* value
AMD	107.61	7.08	0.0001*
Control	50.42		
Dry	49.97	1.56	0.117
Wet	60.96		
Minimal classic	15.29		
Predominant classic	35.75	2.272	0.022*
Occult	30.31	2.270	0.023*
Avastin treated	43.71	0.626	0.532
Not treated	40.21		
Alcoholic	60.57	0.569	0.570
Nonalcoholic	56.78		
Smokers	59.26	0.355	0.722
Nonsmokers	57.03		
Vegetarian	60.13	0.729	0.466
Nonvegetarian	55.59		
Hypertensive	65.13	2.437	0.015*
Nonhypertensive	50.07		
Heart disease	93.62	3.107	0.002*
No heart disease	62.16		
Male	62.80	2.114	0.035*
Female	49.00		

*Significant.
